# Caspase-11 promotes high-fat diet-induced NAFLD by increasing glycolysis, OXPHOS, and pyroptosis in macrophages

**DOI:** 10.3389/fimmu.2023.1113883

**Published:** 2023-01-26

**Authors:** Charles Drummer, Fatma Saaoud, Nirag C. Jhala, Ramon Cueto, Yu Sun, Keman Xu, Ying Shao, Yifan Lu, Huimin Shen, Ling Yang, Yan Zhou, Jun Yu, Sheng Wu, Nathaniel W. Snyder, Wenhui Hu, Jia ‘Joe’ Zhuo, Yinghui Zhong, Xiaohua Jiang, Hong Wang, Xiaofeng Yang

**Affiliations:** ^1^ Centers of Cardiovascular Research, Temple University Lewis Katz School of Medicine, Philadelphia, PA, United States; ^2^ Department of Pathology and Laboratory Medicine, Temple University Lewis Katz School of Medicine, Philadelphia, PA, United States; ^3^ Metabolic Disease Research and Thrombosis Research Center, Departments of Cardiovascular Sciences, Temple University Lewis Katz School of Medicine, Philadelphia, PA, United States; ^4^ Department of Medical Genetics and Molecular Biochemistry, Temple University Lewis Katz School of Medicine, Philadelphia, PA, United States; ^5^ Biostatistics and Bioinformatics Facility, Fox Chase Cancer Center, Temple Health, Philadelphia, PA, United States; ^6^ Tulane Hypertension & Renal Center of Excellence, Tulane University School of Medicine, New Orleans, LA, United States; ^7^ School of Biomedical Engineering, Science and Health Systems, Drexel University, Philadelphia, PA, United States

**Keywords:** non-alcoholic fatty liver disease (NAFLD), non-alcoholic steatohepatitis (NASH), caspase-11, inflammation, pyroptosis

## Abstract

**Introduction:**

Non-alcoholic fatty liver disease (NAFLD) has a global prevalence of 25% of the population and is a leading cause of cirrhosis and hepatocellular carcinoma. NAFLD ranges from simple steatosis (non-alcoholic fatty liver) to non-alcoholic steatohepatitis (NASH). Hepatic macrophages, specifically Kupffer cells (KCs) and monocyte-derived macrophages, act as key players in the progression of NAFLD. Caspases are a family of endoproteases that provide critical connections to cell regulatory networks that sense disease risk factors, control inflammation, and mediate inflammatory cell death (pyroptosis). Caspase-11 can cleave gasdermin D (GSDMD) to induce pyroptosis and specifically defends against bacterial pathogens that invade the cytosol. However, it’s still unknown whether high fat diet (HFD)-facilitated gut microbiota-generated cytoplasmic lipopolysaccharides (LPS) activate caspase-11 and promote NAFLD.

**Methods:**

To examine this hypothesis, we performed liver pathological analysis, RNA-seq, FACS, Western blots, Seahorse mitochondrial stress analyses of macrophages and bone marrow transplantation on HFD-induced NAFLD in WT and Casp11–/– mice.

**Results and Discussion:**

Our results showed that 1) HFD increases body wight, liver wight, plasma cholesterol levels, liver fat deposition, and NAFLD activity score (NAS score) in wild-type (WT) mice; 2) HFD increases the expression of caspase-11, GSDMD, interleukin-1β, and guanylate-binding proteins in WT mice; 3) Caspase-11 deficiency decreases fat liver deposition and NAS score; 4) Caspase-11 deficiency decreases bone marrow monocyte-derived macrophage (MDM) pyroptosis (inflammatory cell death) and inflammatory monocyte (IM) surface GSDMD expression; 5) Caspase-11 deficiency re-programs liver transcriptomes and reduces HFD-induced NAFLD; 6) Caspase-11 deficiency decreases extracellular acidification rates (glycolysis) and oxidative phosphorylation (OXPHOS) in inflammatory fatty acid palmitic acid-stimulated macrophages, indicating that caspase-11 significantly contributes to maintain dual fuel bioenergetics—glycolysis and OXPHOS for promoting pyroptosis in macrophages. These results provide novel insights on the roles of the caspase-11-GSDMD pathway in promoting hepatic macrophage inflammation and pyroptosis and novel targets for future therapeutic interventions involving the transition of NAFLD to NASH, hyperlipidemia, type II diabetes, metabolic syndrome, metabolically healthy obesity, atherosclerotic cardiovascular diseases, autoimmune diseases, liver transplantation, and hepatic cancers.

## 1 Introduction

Nonalcoholic fatty liver disease (NAFLD) is the most common form of chronic liver disease in the Western world. 10% to 40% of adults in the United States are estimated to have some form of NAFLD (1–3). NAFLD is an umbrella term referring to two underlying conditions: nonalcoholic fatty liver (NAFL; also known as hepatic steatosis) and nonalcoholic steatohepatitis (NASH) (4), the inflammatory phase of the disease. Inflammatory liver damage associated with NASH can lead to liver cirrhosis, liver failure, and hepatocellular carcinoma (1, 5–8). NAFLD is considered the hepatic manifestation of metabolic syndrome and metabolically healthy obesity (9–12). In addition, NAFLD is associated with adverse cardiovascular events and contributes to subclinical atherosclerosis (13, 14). Histologically, NAFLD is different from the alcoholic liver disease in at least 11 histologic features results from lipid metabolism imbalance leading to the accumulation of fatty acids in hepatocytes (15, 16). Changes in fatty acid uptake, fatty acid synthesis, lipolysis, β-oxidation and circulating lipoprotein result in hepatocytes exceeding their capacity to safely store lipids (16). These changes lead to the accumulation of toxic lipid species (ceramides, diacylglycerol, lipid peroxides, and oxidized phospholipids) and the subsequent proinflammatory, lipotoxicity-induced hepatocyte cell death (17).

The liver has the highest number of macrophages of any solid organ, which are classified as Kupffer cells (KCs) and monocyte derived macrophages (MDMs) (18–20). Macrophages play a critical role in the initiation and propagation of inflammation in both patients and animal models of NAFLD/NASH (21–25). This is illustrated by the fact that the depletion of KCs is sufficient in halting the NAFL to NASH transitions and preventing the recruitment of bone marrow generated MDMs significantly decreases inflammation associated with NASH (21, 26). Microenvironmental stimuli ultimately determine the function and phenotypic characteristics of the two macrophage subsets and both populations are involved in the development of NAFLD (27–30). Both KCs and MDMs can further differentiate into proinflammatory type 1 (M1) macrophages, which are the primary sources of proinflammatory cytokine secretion and generators of reactive oxygen species (ROS) (31–36) or anti-inflammatory and inflammation resolution type 2 (M2) macrophages (26). True differentiation/polarization of these populations requires single cell transcriptomic analysis of key mediators (22, 23). Therefore, this study will focus on both populations and referred to them collectively as “hepatic macrophages (HMΦs)”. KCs are liver tissue-resident macrophages and reside within liver acinus zone 1 (22, 23). Activated KCs in NAFLD/NASH patients are F4/80^+^, CD14^+^, CD16^+^ and CD68^+^ (21, 27, 37–40) and in NAFLD mouse models are F4/80^+^, CD11b^low^, CD68^+^ and Clec4f^+^ (21, 27, 38). KCs have several homeostatic functions including clearance of damaged red blood cells, iron metabolism, bilirubin metabolism, and cholesterol metabolism (18, 19, 31, 41–43).

MDMs arise from Ly6C^+/high^ bone marrow (BM)-derived monocytes recruited to the liver in response to liver inflammation (21–23). Upon maturation, MDMs transition to a Ly6C^-/low^ status and reside in hepatic acinus zones 2 and zone 3 (22, 23, 44, 45). These MDMs repopulate the liver macrophage niche in hepatic acinus zone 1, with the microenvironment and secretomes from liver sinusoidal endothelial cells (LSECs) allowing for the development of a “KC-like” phenotype and functionality (27, 29, 30, 38). Recent studies have added more context to this binary system (46). Hepatic macrophages play a significant role in NAFLD (21, 47–50). MDMs are a population of HMΦs capable of repopulating the liver when KCs are depleted in chronic liver diseases. Furthermore, clinical trials have demonstrated that preventing MDM infiltration decreases inflammatory liver damages (21, 23, 27, 29, 30, 38, 50–54).

Inflammasomes and inflammatory caspases, such as caspase-1 and caspase-4 (humans)/caspase-11 (mice), have two coupled functions: 1) serve as sensors for danger associated molecular patterns (DAMPs) and viral and bacterial infections-related pathogen associated molecular patterns (PAMPs) and 2) initiate inflammation signaling and promote inflammatory cell death (pyroptosis) (55–70), which specifically antagonize infection but can cause detrimental inflammation as well (71). Recent studies have shown promise in treating NAFLD with therapeutics targeting the nucleotide-binding domain (NOD)-, leucine-rich repeat (LRR)- and pyrin domain-containing protein 3 (NLRP3) inflammasome, suggesting that targeting pyroptosis is a viable option for treating the disease (72, 73). While current pyroptosis targeting therapeutics focus on the inflammasomes that activate caspase-1, the caspase-11-dependent pathway presents a novel therapeutic target. Our group and others have demonstrated that caspase-1 deficiency is protective against HFD-induced NAFLD to NASH progression (57, 74). However, the roles of caspase-11-dependent pathway have not been defined in HFD-induced NAFLD (75, 76).

As we recently published in our novel data mining analysis of 4249 genes in 27 mouse models of NAFLD, caspase-11 mediates liver pyroptosis in both patients and mouse models of NASH *via* lipid peroxidation and trained immunity (innate immune memory) pathways (77). Other studies have demonstrated that lipid peroxidation can generate endogenous ligand for caspase-11, which impacts animal models of sepsis (78–80).

Endotoxin levels and gut-derived Gram-negative bacteria are elevated in patients with NAFLD (81). A previous study involving two obese individuals showed that the nonvirulent endotoxin-producing strains of pathogenic species that were overgrowing in obese people’s guts can cause NAFLD and related metabolic problems. The most upstream and crucial biological process that causes all phenotypes in NAFLD and other related metabolic disorders is the host’s TLR4 receptor (82). Another study involved Nine hundred and twenty adults randomly selected from the government’s census database and underwent proton-magnetic resonance spectroscopy to assess hepatic steatosis showed that NAFLD patients had slightly higher lipopolysaccharide-binding protein (LBP) endotoxin markers associated with insulin resistance and dyslipidemia and that people with modest alcohol consumption have lower serum endotoxin (83). Similarly, another study involving one hundred and fifty-five patients with NAFLD and twenty-three control individuals showed that endotoxin levels were significantly higher in NAFLD patients than in controls, with particularly noticeable increases in early-stage fibrosis (84). However, several important questions remain to be addressed: 1) Why NAFLD cannot be developed in germ-free mice; and why LPS-containing Gram-negative bacteria overgrowing in human gut microbiota are linked to NAFLD (82). 2) Why NAFLD can be a significant proinflammatory driver for second wave of atherosclerosis (11, 12). We previously reported a novel metabolically healthy obesity mouse model, in which atherosclerosis is decreased due to proinflammatory microRNA-155 (miR155) deficiency in apolipoprotein E deficient (ApoE^–/–^) mice but NAFLD development is sustained (10). 3) Why HFD model becomes an essential component for all 27 models of NAFLD. Here we sought to determine whether caspase-11 plays a role in promoting HFD-induced NAFLD. We found that HFD feeding for 12 weeks drives NAFLD in WT mice, which are transcriptionally distinct from WT liver fed with normal chow diet (NCD). HFD increased gene expressions of caspase-11, gasdermin D (GSDMD), interleukin-1β (IL-1β), and guanylate-binding proteins (GBPs) in liver. However, caspase-11 deficiency significantly decreased liver IL-1β concentrations, reduced N-terminal GSDMD expression on plasma membrane, significantly re-programed liver transcriptomes, and attenuated hepatic monocyte/macrophage pyroptosis in HFD-induced NAFLD. BM-derived monocytes/macrophages play more significant roles than liver resident monocytes/macrophages in developing pyroptosis. To determine the underlying mechanisms, we performed a set of experiments and found that caspase-11 deficiency significantly decreased extracellular acidification rate (ECAR) from glycolysis and oxidative phosphorylation (OXPHOS), indicating that caspase-11 significantly contributes to maintain dual fuel bioenergetics — glycolysis and OXPHOS in proinflammatory fatty acid palmitic acid-stimulated macrophages, and potentially promotes transition of M2 macrophages into M1 macrophages. These results provide novel insights on the roles of caspase-11-GSDMD pathway in promoting hepatic macrophage inflammation and novel targets for future therapeutic interventions involving transition of NAFLD to NASH, hyperlipidemia, type-II diabetes, metabolic syndrome, atherosclerotic cardiovascular diseases, autoimmune diseases, liver transplantation, and hepatic cancers.

## 2 Materials and methods

### 2.1 Animal care

All animal experiments were performed in accordance with the Institutional Animal Care and Use Committee (IACUC) guidelines and were approved by the IACUC of Lewis Katz School of Medicine (LKSOM) at Temple University. Wild-type (WT) mice were of a C57BL/6J background, and caspase-11 knockout (Casp11^–/–^) mice were purchased from Jackson Laboratories (Bar Harbor, ME). Mice were housed under controlled conditions in the LKSOM Animal Facility, where they had *ad libitum* access to standard chow diet control/HFD, water, and were subject to a 12-hour light-dark cycle. Mice were age-matched and gender-specific in all experiment groups. At eight to ten weeks old, male mice either remained on normal chow diet (10.7% fat, 23.9% protein, 5.1% fiber, 58.7% carbohydrate/other, 200ppm cholesterol; Labdiet 5001) or switched to HFD [20% (w/w) fat, 17.4% protein, 5% fiber, 49.9% carbohydrate/other, 2027 ppm cholesterol (0.15% (w/w) cholesterol); AIN-76A] (Research Diets, NJ).

### 2.2 Histological NAFLD activity score analysis

There are different parameters used to histologically grade NAFLD/NASH progression including: macrovesicular steatosis, microvesicular steatosis, lobular inflammation, Mallory Body occurrence, hepatocellular iron, KC activation, and hepatocyte ballooning (1, 85, 86). The most common NAFLD/NASH grading rubric is the “NAFLD Activity (NAS) Score” which combines (macrovesicular) steatosis, lobular inflammation, and hepatocyte ballooning on a scale from 0 (no NAFLD) to 8 (severe disease) (1, 85, 86). Mice were sacrificed *via* ketamine overdose and cervical dislocation. Body weight was measured then mice were affixed to a Styrofoam surface. Blood was collected *via* cardiac puncture and the liver was perfused *via* the portal vein with 10 ml of phosphate buffered saline (PBS). Isolated liver was weight, washed with PBS. A liver sample was collected and preserved in 10% formalin for 8 – 10 hours at room temperature (RT), washed with PBS then stored in 75% ethanol. Hematoxylin and eosin staining was carried out by AML Laboratories (St. Augustine, FL). NAFLD Activity Score (NAS) was determined by Dr. Nirag Jhala, MD (Professor, Pathology and Laboratory Medicine) from Temple University Hospital (Philadelphia, PA).

### 2.3 Plasma cholesterol measurement

Blood was collected in 5% coated tubes *via* from the cardiac puncture of anesthetized animals. Plasma was collected by low-speed centrifugation for 20 minutes at 4°C. Plasma cholesterol levels in each sample were analyzed at the Mouse Metabolic Phenotyping Center at the University of Cincinnati (C1052-lipid Profiles) by colorimetric assays using Cholesterol Reagent Set. Reactions were run in microtiter plates and analyzed on a plate reader (https://med.uc.edu/institutes/mmpc/select-a-test/lipid-metabolism).

### 2.4 Bone marrow transplantation

Six to eight weeks old recipient (male, CD45.2^+^) mice were irradiated with 750 to 950 cGy (RS-2000 Biological Irradiator, Buford, GA). Eight to ten weeks old donor (male, CD45.1^+^) mice were sacrificed as described above. After euthanasia, femur and tibia were amputated and stored in PBS on ice. Marrow was flushed from the bone, passed through a 70-um filter. Red blood cells were eliminated using erythrocyte lysis buffer (8.29 g/L NH_4_Cl, 1 g/L KHCO_3_, 37.2 mg/L EDTA, double-distilled H_2_O, pHed to pH 7.2). 1x10^6^ cells from donor mice were transplanted by retro-orbital injection into recipient mice.

### 2.5 Liver single cell suspension and immune cell fractionation

Mice were sacrificed, and liver was perfused *via* the portal vein with 10 ml of PBS. Isolated liver was weight, cut into 1-1.5 mm pieces and stored in 5 ml Liver Digestion Medium (ThermoFisher, 17703034) on ice until ready for digestion. Liver suspensions were incubated in a 37 °C water bath for 20-30 minutes on an orbital shaker. Digestion medium was collected in 5.0 ml Eppendorf tubes for cytokine analysis. Liver suspensions were filtered through a 70-um filter. Red blood cells were eliminated using erythrocyte lysis buffer. HMΦs were separated from hepatocytes using 33% Percoll solution (Sigma-Aldrich, P1644).

### 2.6 Western blot and ELISA analysis

Immune cell fraction was prepared from mouse liver as described above. Fractionated immune cells were lysed and protein isolated using an acid-guanidinium-phenol based reagent TRIzol (ThermoFisher, GE17-0891-01) according to manufacturer’s instructions. Protein was concentrated using Protein Concentrator polyethersulfone (PES), 10K molecular weight cutoff (MWCO) (ThermoFisher, 88503). Protein was quantified using colorimetric Pierce BCA Protein Assay Kit (ThermoFisher 23225). Protein was run on 12.5% gel and transferred to polyvinylidene difluoride (PVDF) membrane. Membranes were blocked using 5% bovine serum albumin (BSA) for 1 hour. Primary antibodies were diluted in Tris-buffered saline (TBS) (0.2% Tween-20) buffer and incubated at 4°C overnight. Primary antibodies used: caspase-11 (ThermoFisher, 14-9935-82), GSDMD (Abcam, ab209845), and β-Actin (Sigma-Aldrich, ab6276). Secondary antibodies were diluted in TBS (0.2% Tween-20, 0.01% SDS) and incubated for 30 minutes to 1 hour at RT. Secondary antibody used: IRDye 680RD (LI-COR), IRDye 800CW (LI-COR). Membranes were scanned using LI-COR Odyssey Crx (Li-Cor Biosciences, Lincoln, NE). Image processing was performed using Image Studio Analysis (LI-COR). Liver IL-1β levels were assessed using the Mouse IL-1 beta/IL-1F2 Quantikine ELISA Kit (R&D Systems, MLB003) following the manufacturer’s instruction.

### 2.7 Flow cytometric quantification of hepatic macrophages and noncanonical pyroptosis

Animals were sacrificed and livers were collected as described above. Fractionated immune cells were collected. Cells were incubated with CD16/CD32 FcR-blocking antibody (BD Bioscience, 553142) on ice for 20 minutes. Live/Dead staining was performed using Zombie Aqua (Biolegend, 423101) with a 30-minute incubation on ice. HMΦ surface staining was performed using the following panel: APC-Cy7_CD45 (BioLegend, 103116), FITC_I-A/I-E (MHCII) (BioLegend, 107605), PerCP-Cy5.5_CD11b (BioLegend, 101228), BV510_Ly6G (BioLegend, 127633), BUV395_F4/80 (BD Biosciences, 565614), BV421_CCR2 (BioLegend, 150605), APC_Ly6C (BioLegend, 128016), PE-Cy7_CD206 (ThermoFisher, 25-2061-82), BV785_CD86 (BioLegend, 105043). HMΦ pyroptosis was performed using the following panel: FAM-LEHD-FMK (caspase-11 activity assay), APC-Cy7_CD45 (BioLegend, 103116), BV510_Ly6G (BioLegend, 127633), PerCP-Cy5.5_CD11b (BioLegend, 101228), Ly-6C_AF700 (BioLegend, 128024), BUV395_F4/80 (BD Biosciences, 565614), GSDMDC1_AF674 (Santa Cruz Biotechnology, sc-393581 AF647). Flow cytometric data was acquired using LSR-II Flow Cytometer (BD Bioscience). Mean fluorescent intensity (MFI) and population percentages were analyzed using FlowJo (Ashland, OR).

### 2.8 RNA-sequencing

RNA-seq was performed using the immune cell fraction from male WT and Casp11^–/–^ mice fed 12-week HFD. Animals were sacrificed and livers were collected. Fractionated immune cells were lysed and RNA isolated using TRIzol (ThermoFisher, GE17-0891-01) according to manufacturer’s instructions. RNA was quantified using Nanodrop (ThermoFisher). Frozen RNA samples were sent to Genewiz (South Plainfield, NJ) for RNA-seq analysis. Total RNA libraries were prepared by using Pico Input SMARTer Stranded Total RNA-seq Kit (Takara). In short, 10 ng total RNA from each sample was reverse transcribed *via* random priming and reverse transcriptase. Full-length cDNA was obtained with SMART (Switching Mechanism At 5′ end of RNA Template) technology. The template-switching reaction was used to keep the strand orientation of the RNA. The ribosomal cDNA was hybridized to mammalian-specific R-Probes and then cleaved by ZapR. Libraries containing Illumina adapter with TruSeq HT indexes were subsequently pooled and loaded to the Hiseq 2500. Single end reads at 75 bp with 30 million reads per sample were generated for bioinformatic analysis FASTQ files were mapped to the mouse mm10 genome using STAR Aligner and BAM alignment files were imported into Qlucore Omics Explorer and used to generate expression data (transcripts per million, TPM). All original RNA-seq data were deposited in the NCBI’s Gene Expression Omnibus database (GSE221005).

### 2.9 Measurement of extracellular acidification rate and mitochondrial parameters in palmitic acid stimulated bone marrow-derived macrophages

Bone marrow-derived macrophages were isolated from tibias and femurs of WT and Casp11^–/–^ as we previously reported (12). Briefly, femurs and tibias were sprayed with 75% alcohol in Petri dish containing Roswell Park Memorial Institute medium (RPMI) 1640 (Gibco, Grand Island, NY) with 2% FBS. The bones were cut off at both ends and elute morrow into 50-mL conical tubes with RPMI-1640 with 2% FBS and penicillin/streptomycin (p/s) (Gibco, Grand Island, NY) using 10-mL syringes and 25-G needles. The cell suspension was filtered through a 70-μm cell strainer (BD Biosciences, San Jose, CA) into a sterile conical tube and centrifuged (500 g for 5 minutes). The pellet was resuspended well in 5 to 10 mL ACK red blood cell lysis buffer (Sigma-Aldrich, St Louis, MO) for 1 minute followed by the addition of RPMI-1640 and centrifugation (600 g for 7 minutes) to terminate the lysis. The pellet was washed using RPMI-1640 with 10% FBS once more, resuspended in differentiation medium (RPMI-1640, 10% FBS, 20% L929 conditional medium, p/s), and cultured at 37℃ in a 5% CO2 incubator. At day 3, the supernatant was carefully removed, and the medium was replaced. At day 7, cells were harvested and transferred to 96-well plate for seahorse assay. Seahorse XF96 analyzer (Seahorse Bioscience, Agilent, Santa Clara, CA) was used to measure the extracellular acidification rate (glycolysis) and six mitochondrial parameters (Mito Stress Test) in bone marrow-derived macrophages, including basal respiration, maximal respiration, proton leak, ATP production, spare respiratory capacity, and non-mitochondrial respiration as we previously reported (87, 88). Briefly, 100,000 cell/well were seeded in a 96-well plate and cultured overnight in XF assay medium supplemented with 10 mM glucose, 1 mM pyruvate and 2 mM L-glutamine. Cells were stimulated with palmitic acid 500 µM for 8 hours (89). Culturing media was changed to modified DMEM media and placed into a 37°C non-CO2 incubator for 1 hour. After preparation of drugs and XF Cell Mito Stress Test Kit and glycolytic rate kit (Seahorse Bioscience) into cartridge ports, the cartridge and cell culture plates were loaded into XF96 analyzer (Seahorse Bioscience). Experiments were performed in triplicates.

### 2.10 Statistical analysis

All data was reported as mean ± standard deviation (SD). Statistical analysis comparing genotype-diet groups (WT-HFD versus (vs) WT-NCD, Casp11^–/–^HFD vs Casp11^–/–^NCD, Casp11^–/–^NCD vs WT-NCD) were calculated by 1-way or 2-way ANOVA using Prism (GraphPad) or Qlucore Omics Explorer (Qlucore). Statistical significance was set at *p* ≤ 0.05.

## 3 Results

### 3.1 High-fat diet promotes nonalcoholic fatty liver disease, and lipid accumulation are temporally earlier than liver inflammation

**Figure 1:**
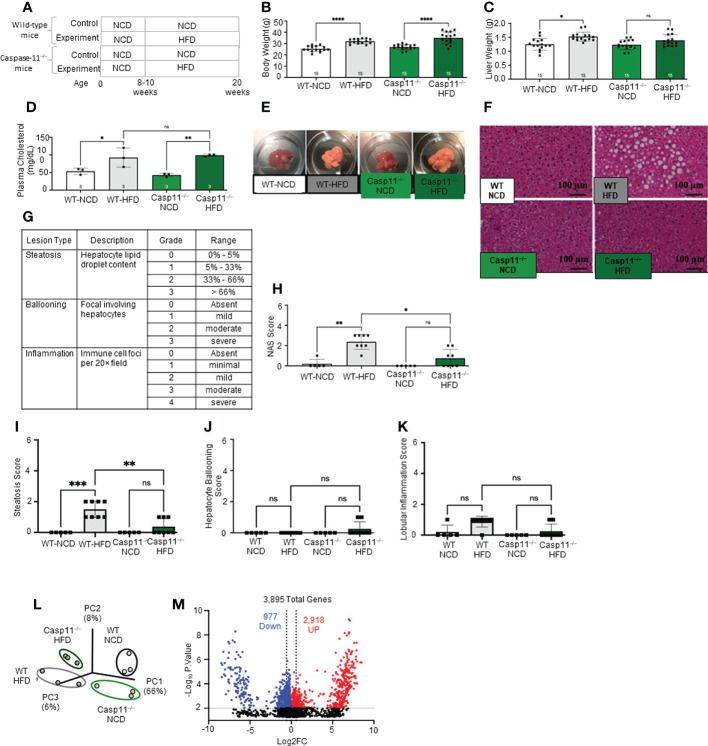
High-fat diet (HFD) promotes non-alcoholic fatty liver disease (NAFLD) and Caspase-11 deficiency decreases lipid droplet, steatosis score, and NAS score in HFD-induced NAFLD but does not change liver weight or gross anatomic fatty liver pathology. **(A)** Experimental diet timeline. 8-10 weeks old Wild-type (WT) male mice and Caspase-11 deficient (Casp11–/–) mice were fed HFD or normal chow diet (NCD) for 12 weeks. **(B)** Body weight (n = 15). **(C)** Liver weight (n = 15). **(D)** Plasma cholesterol levels (n = 3). **(E)** Representative images showed that HFD feeding changed liver color to light yellow color. **(F)** Representative 20X images of hematoxylin and eosin (H&E) staining showing hepatic steatosis in 12-week HFD-fed WT and Casp11–/– male mice compared to 12-week NCD. Scale bar 100 µm. **(G)**. Description and grades of NAS scare. NAFLD activity score analysis indicated that lipids accumulation as judged by liver steatosis and ballooning is precedent to liver inflammation. In grade 1, when steatosis reaches 5-33%, and ballooning reaches mild, inflammation is minimal. Steatosis was in the graded section as: are 0, steatotic; 0% to 1, 5% greater than 5% to 33% of hepatocytes are steatotic; 2, greater than 33% to as: 66%; 0, absent; and 3, 1, greater mild (focal; than 66%. involving Ballooning fewer than 3 hepatocytes); 2, moderate (focal and involving 3 or more hepatocytes or multifocal); and 3, severe (multifocal, with more than 2 foci of 3 or more hepatocytes). (0 or 1 Inflammation focus per 20× was field); graded 2, as: mild 0, (2 absent; foci); 3, moderate (3 foci); and 4, severe (4 or more foci per 20× field). **(H)** Total NAS score for WT-NCD (n = 5), WT-HFD (n = 8), Casp11–/–NCD (n = 5) and Casp11–/–HFD (n = 8). **(I)** Hepatic steatosis score. **(J)** Hepatocyte ballooning. **(K)** Lobular inflammation. **(L)** Principal component analysis (PCA) demonstrating that WT-NCD, WT-HFD, Casp11–/–NCD, and Casp11–/–HFD mice are transcriptionally distinct (n = 3). **(M)** Volcano plot analysis showed the 3895 differentially expressed genes (DEGs) in the WT liver of 12-week HFD compared to12-week NCD control. Among 3895 DEGs, 2918 genes were significantly upregulated (red), and 977 genes downregulated (blue). (FC) > 1.5 and p < 0.05. Statistical Analysis: Bulk RNA-Seq analysis was performed using Qlucore Omics Explorer. PCA plot generated using significantly differentially regulated genes). Volcano plot generated using GraphPad Prism with significantly differentially regulated genes. Statistical Analysis: One-Way ANOVA. *p < 0.05, **p < 0.001, ***p < 0.0001 ****p < 0.0001. ns, Non-significant.

HFD feeding is a commonly used rodent model of Western diet-induced obesity and NAFLD (90–92). The HFD not only increases intake of saturated fatty acid (SFA) but also induces metabolic endotoxemia, defined as an HFD-associated increase in circulating LPS (93). The 12- and 16-week HFD-feeding schemes have been shown to induce obesity and NAFLD in male C57BL/6 mice (94). Therefore, we compared WT mice fed either HFD or normal chow diet (NCD) (as controls) for 12 weeks (**Figure 1A**). We found that HFD significantly increased mouse body weight (**Figure 1B**), liver weigh (**Figure 1C**), and plasma cholesterol levels (**Figure 1D**). HFD for 12 weeks has been shown to discolor the liver and increase macrovesicular steatosis (1, 85, 86). Our results also showed that the liver color of the HFD group appeared light yellow compared to dark red in the NCD group (**Figure 1E**), consistent with the HFD promoted hepatic steatosis.

After 12 weeks of HFD, there was a NAFLD/NASH histological phenotype with deposits of fat determined by pathohistological staining (**Figure 1F**). The NAFLD activity score (NAS) is a pathological measure of grade and represent the sum of scores for steatosis (0-3), hepatocyte ballooning (0-3), and lobular inflammation (0-3). In grade 0, steatosis less than 5%, no hepatocyte ballooning, and no inflammation. Grade 1 showed mild steatosis (5-33%), mild hepatocyte ballooning, and minimal inflammation. Grade 2 has moderate steatosis (33-66%), moderate hepatocyte ballooning, and mild inflammation. Grade 3 has severe steatosis (> 66%), severe hepatocyte ballooning, and moderate inflammation. However, grade 4 has severe inflammation (4) (**Figure 1G**). In addition, HFD significantly increased NAS score (**Figure 1H**), steatosis score (**Figure 1I**), and slightly but not statistically significant increase in lobular inflammation (Figure 1K), and no change in hepatocyte ballooning (**Figure 1J**) compared to NCD controls.

Additionally, our new RNA-seq data indicated that the livers of HFD mice were transcriptionally distinct from that of NCD-fed mouse liver controls (**Figure 1L**). As shown in the Volcano plot analysis (**Figure 1M**), HFD modulated the expressions of 3895 genes, among them 2918 genes were significantly upregulated, and 977 gene downregulated (FC ≥ 1.5 and *p* value ≤ 0.01). Taken together, these results have demonstrated that HFD promotes NAFLD phenotypes, and induce gross pathological and transcriptomic changes in the liver characterized by deposits of fat that appear temporally earlier than liver inflammation.

### 3.2 HFD upregulates the expressions of proinflammatory, NASH-related hepatic macrophage markers, guanylate binding proteins, caspase-11, and increases N-terminal gasdermin D (GSDMD) cleavage

**Figure 2:**
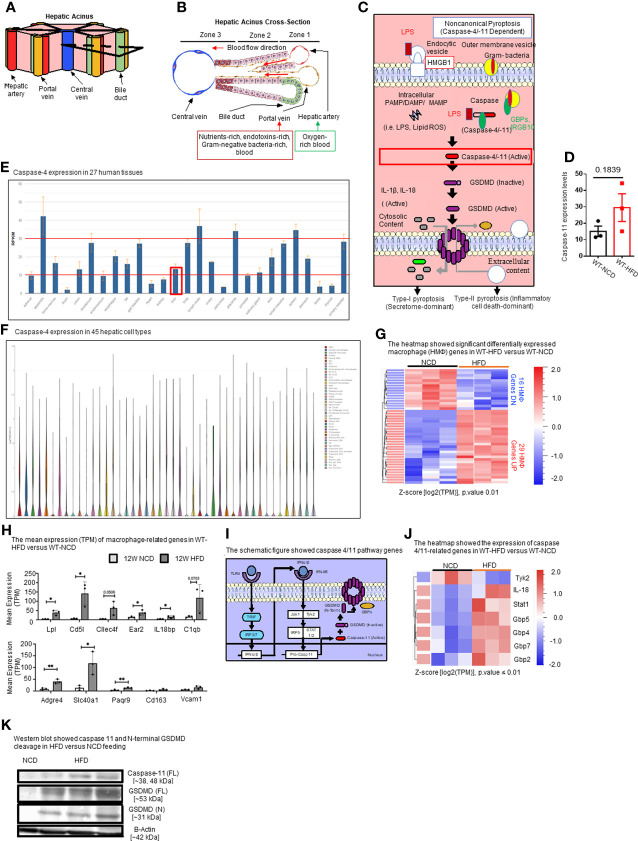
HFD upregulates the expressions of proinflammatory, NASH-related hepatic macrophage markers, GBPs, caspase-11, and increases N-terminal GSDMD cleavage. **(A, B)** The structure of hepatic acinus suggests that the liver is the first organ exposed to endotoxins-rich and Gram bacteria-rich blood in the body. Liver parenchymal and nonparenchymal cells are organized into structures called “acinus”. Hepatic acini form hexagonal structures with a central vein and portal triad at the vertices. The hepatic acinus can be histologically divided into three zones. Zone 1 represents the portal triad which includes a hepatic artery, a portal vein, and a bile duct. Zone 2 represents the parenchymal area, structurally consisting primarily of hepatocytes with a central vasculature composed of liver sinusoidal endothelial cells (LSECs). Mature immune cells including B cells, T cells, innate-like lymphoid cells (ILCs), natural killer cells (NK cells), and tissue-resident macrophages (Kupffer cells) reside in hepatic acinus zone 1 and 2. Zone 3 represents the central vein, the innermost hepatocytes, and infiltrating immune cells. The portal vein contains nutrient-rich, endotoxins-rich, and gram bacteria-rich blood. **(C)** Noncanonical pyroptosis is caspase-4 (human), caspase-11 (mouse) dependent. Guanylate binding proteins (GBPs) promote the outer membrane vesicles from gram-bacteria to activate caspase-11. Intracellular sources of inflammation (including LPS and oxidized phospholipids) directly bind to caspase-4/-11. Caspase-4/-11 cleaves gasdermin-D to initiate noncanonical pyroptosis. **(D)** The expressions of caspase-11 in the liver were in an upregulation trend in high-fat diet-fed wild-type mice. The microarray data were achieved from the NIH-NCBI-Geo-Profiles database (GDS4811). **(E)** Caspase-4 expression in 27 human tissues. The expression of caspase-4 in the liver is at a medium level among all 27 human tissues. Caspase-4 RNA-Seq data were analyzed from the NIH-NCBI-Gene database (https://www.ncbi.nlm.nih.gov/gene/837). **(F)** Caspase-4 expression in 45 hepatic cell types. Caspase-4 is expressed in all 40 immune cell types, the single-cell RNA-Seq data were analyzed from the MIT Broad Institute Single Cell RNA-Seq (scRNA-Seq) Porter database (https://singlecell.broadinstitute.org/single_cell/study/SCP1845/cross-tissue-immune-cell-analysis-reveals-tissue-specific-features-in-humans?genes=casp4%26tab=distribution#study-visualize). Among 45 immune cell types identified in scRNA-Seq, 19 immune cell types are significantly enriched in the human liver including dendritic cell 1 (DC1), DC2, classical monocytes, non-classical monocytes, erythrophagocytic macrophages, mononuclear phagocytes (MNP)/B doublets, age-associated B cells (ABCs), plasma cells, Plasmablasts, MNP/B doublets, T/B doublets, mucosal-associated invariant T (MAIT), T_CD4/CD8, T effector memory (Tem)/effector memory re-expressing CD45RA (emra)_CD8, T resident memory cell/effector memory cell (Trm/em)_CD8, gamma-delta T cell (Tgd)_CRTAM+, Cycling T cell & natural killer cell (NK), NK_CD16+, and NK-CD56bright_CD16-. **(G)** 12-week HFD promotes expression of HMΦ activation mediators in the WT liver. 8-10 weeks old male WT mice were fed with HFD for 12 weeks. Heatmap of significant, differentially regulated macrophage (HMΦ) genes. **(H)** Bulk RNA-seq expression (mean transcripts per kilobase million, TPM) of macrophage mediators. **(I)** Schematic representing caspase-4/11 pathway genes. **(J)** Bulk RNA-seq expression (mean TPM) of noncanonical pyroptosis-associated mediators. **(K)** Western blot analysis showed that HFD feeding increased caspase-11 and N-terminal GSDMD cleavage. Statistical Analysis: Bulk RNAseq analysis was performed using Qlucore Omics Explorer. Heatmap was generated using significantly differentially regulated genes (p.adj < 0.01). Differential gene expression presented as Z-score calculated from log2 transformed TPM. *P < 0.05, **p < 0.01.

Hepatic acini form hexagonal structures with a central vein and portal triads at every other vertices. The hepatic acinus can be histologically divided into three zones (**Figures 2A, B**). Zone 1 represents the portal triad, which includes hepatic artery, portal vein, and bile duct. Zone 2 represents the parenchymal area, structurally consisting primarily of hepatocytes with a central vasculature composed of LSECs. Mature immune cells including B cells, T cells, innate-like lymphoid cells (ILCs), natural killer cells (NK cells), and KCs reside in hepatic acinus zone 1 and 2. Zone 3 represents the central vein, the innermost hepatocytes, and infiltrating immune cells. The portal vein contains nutrient-rich blood that is contaminated by microbial pathogen associated molecular patterns (PAMPs) such as LPS and bacteria, both arising from the intestines (95).

In the noncanonical pyroptosis pathway (**Figure 2C**), the guanylate binding proteins (GBPs) promote exposure of LPS from Gram-negative bacteria to activate caspase-11 (96, 97). Cytosolic LPS then directly binds to and activates caspase-4/11, leading to GSDMD cleavage to generate the N-terminal active fragment (GSDMD-NT). GSDMD-NT-mediated plasma membrane perforation triggers membrane rupture associated with release of proinflammatory cytokines such as IL-1β (type-I pyroptosis or secretome-dominant) and cell death (type-II pyroptosis or inflammatory cell death-dominant) (97–100). Previous studies have shown the role of inflammasomes and caspase-1 in NAFLD (101, 102), and the role of caspase-11 in methionine-, choline-deficient diet (MCD)-induced NASH was reported (76). However, the roles of caspase-11 in HFD-induced NAFLD have not been extensively studied. Therefore, we examined the expression levels of caspase-11 in the liver of HFD-fed WT mice from microarray data from the NIH-NCBI-Geo-Profiles database (GDS4811). The results showed that caspase-11 expression was increased in the liver of HFD fed mice (**Figure 2D**). We further checked the expression of caspase-4 in the normal human tissues from RNA-seq data performed on tissue samples from 95 human individuals representing 27 different tissues, data were analyzed from the NIH-NCBI-Gene database (https://www.ncbi.nlm.nih.gov/gene/837) (103). Our data analysis showed that caspase-4 expression in the liver is at a medium level among all 27 human tissues (**Figure 2E**). Furthermore, we analyzed the expression of caspase-4 in 45 immune cell types from single-cell RNA-seq data collected from the MIT Broad Institute Single Cell RNA-seq (scRNA-seq) Porter database (https://singlecell.broadinstitute.org/single_cell/study/SCP1845/cross-tissue-immune-cell-analysis-reveals-tissue-specific-features-in-humans?genes=casp4&tab=distribution#study-visualize) (104). Our data analysis showed that caspase-4 was expressed in all 45 immune cell types (**Figure 2F**). Among 45 immune cell types identified in scRNA-seq, 19 immune cell types are significantly enriched in the human liver including dendritic cell 1 (DC1), DC2, classical monocytes, non-classical monocytes, erythrophagocytic macrophages, mononuclear phagocytes (MNP)/B doublets, age-associated B cells (ABCs), plasma cells, plasmablasts, MNP/B doublets, T/B doublets, mucosal-associated invariant T (MAIT), T_CD4/CD8, T effector memory (Tem)/effector memory re-expressing CD45RA (emra)_CD8, T resident memory cell/effector memory cell (Trm/em)_CD8, gamma-delta T cell (Tgd)_CRTAM+, Cycling T cell & natural killer cell (NK), NK_CD16+, and NK-CD56bright_CD16.

Liver has the highest number of macrophages of any solid organ (18–20), therefore, we focused on inflammatory features of liver macrophages to determine the inflammatory pathways underlying HFD-driven NAFLD transition to NASH. Our RNA-seq data analysis of liver immune cells showed that HFD increased the expression of fifteen NASH-associated inflammatory macrophage markers including fatty acid binding protein 7 (Fabp7), C-C motif chemokine ligand 24 (Ccl24), lipoprotein lipase (Lpl), matrix metallopeptidase 12 (Mmp12), complement C1q B chain (C1qb), interleukin 18 binding protein (Il18bp), C-type lectin domain family 4 member F (Clec4f), CD5 molecule like (Cd5l), phospholipid transfer protein (Pltp), nuclear receptor subfamily 2 group F member 6 (NR2F6, Ear2), insulin like growth factor 1 (Igf1), apolipoprotein C1 (Apoc1), WAP four-disulfide core domain 17 (Wfdc17), membrane spanning 4-domains A7 (Ms4a7), and matrix metallopeptidase 12 (Mmp12) (**Figures 2G, H**). Ten healthy, inactivate HMΦ markers were also increased in HFD including macrophage receptor with collagenous structure (Marco), C-X-C motif chemokine ligand 13 (Cxcl13), CD163 molecule (Cd163), adhesion G protein-coupled receptor E4, pseudogene (Adgre4), progestin and adipoQ receptor family member 9 (Paqr9), solute carrier family 40 member 1 (Slc40a1), ficolin 3 (Fcna), mannose receptor C-type 1 (Mrc1), syndecan 3 (Sdc3), and heme oxygenase 1 (Hmox1), likely signifying an expansion of KCs in preparation for HFD-induced activation (**Figures 2G, H**). In addition, the expressions of other genes in the caspase-11 pathway, shown in schematic **Figure 2I** including signal transducer and activator of transcription 1 (Stat1), guanylate binding protein 2 (Gbp2), Gbp4, Gbp5, Gbp7, and interleukin-18 (IL-18) were significantly increased in the livers of HFD fed mice (**Figure 2J**). Western blot analysis also showed that caspase-11 and GSDMD-NT protein expressions were increased by HFD (**Figure 2K**). Tyrosine kinase 2 (Tyk2) as a part of Janus kinase (JAK)-signal transducer and activator of transcription (STAT) (JAK/STAT) signaling downstream of interferon-α/β receptor (IFNAR) has been shown to increase caspase-11 expression in splenic myeloid cells in response to LPS stimulations (105). IL-18 is the other IL-1 family cytokine cleaved by proinflammatory caspases in pyroptosis (102). The N-terminal of cleaved GSDMD is required for GSDMD-pore formation on plasma membrane and demonstrates caspase-11 activity. Guanylate-binding protein (GBP) expression have been shown to bind to cytosolic Gram-negative bacteria and expose LPS for sensing by caspase-11 (106). Taken together, these results have demonstrated that HFD upregulates the expressions of proinflammatory, NASH-related hepatic macrophage markers, GBPs, caspase-11, GSDMD, and increases GSDMD-NT cleavage and membrane expression.

### 3.3 Caspase-11 deficiency decreases lipid droplet, steatosis score, and non-alcoholic steatosis score in HFD-induced NAFLD but does not change liver weight or gross anatomic fatty liver pathology

To determine the roles of caspase-11 in NAFLD, we compared pathological progression of NAFLD in Casp11^–/–^mice with that of WT control mice (**Figure 1A**). We found that HFD significantly increased body weight for both Casp11^–/–^ and WT mice (**Figure 1B**). While liver weight significantly increased in HFD fed WT mice, the liver weight of Casp11^–/–^ mice did not increase significantly (**Figure 1C**). However, HFD promoted steatosis and significantly increased circulating cholesterol levels in both Casp11^–/–^ and WT mice (**Figures 1D**). Along these lines, HFD increased lipid droplet formation (macrovesicular steatosis), NAS score and steatosis score (107) in WT mice. However, HFD in Casp11^–/–^ mice dramatically reduced lipid droplet, NAS score, and steatosis score compared to WT mice on HFD (**Figures 1E, I**) and slightly but statistically non-significant decreased lobular inflammation (**Figure 1K**). Interestingly, caspase-11 deficiency led to slightly but statistically non-significant increases in hepatocyte ballooning (swollen hepatocytes with rarefied cytoplasm) (108), which was not seen in any of the other groups (**Figure 1J**). Decreased steatosis and increased hepatocyte ballooning suggest differential roles for caspase-11 in both hepatocytes and HMΦs, respectively. Our principal component analysis (PCA) of liver RNA-seq data showed that liver transcriptomes of WT and Casp11^–/–^ mice on HFD were transcriptionally distinct from the respective NCD controls (**Figure 1L**). In summary, our results have demonstrated that although slightly increasing hepatocyte ballooning, caspase-11 deficiency decreases NAFLD progression and lobular inflammation in HFD-induced NAFLD.

### 3.4 Caspase-11 deficiency reprograms liver transcriptomes and attenuates hepatic macrophage pyroptosis in HFD-induced NAFLD; caspase-11 cleaves N-terminal GSDMD in normal chow diet livers more than that in HFD-induced NAFLD; and bone marrow-derived macrophages play more significant roles than liver resident macrophages in facilitating pyroptosis

**Figure 3:**
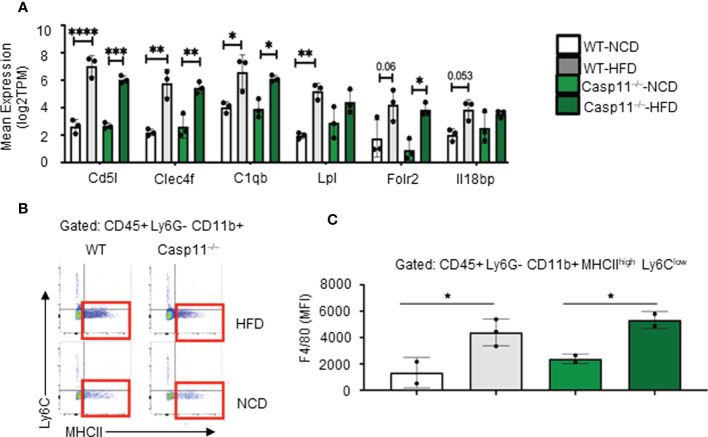
High-fat diet (HFD)-induced non-alcoholic fatty liver disease (NAFLD) drives the increase of NASH-related F4/80+ hepatic macrophages (HMΦs) in WT mice, which are caspase-11 activation-independent. 8-10 weeks old male WT and Casp11^–/–^ mice were fed HFD for 12 weeks. **(A)** Bulk RNA-seq expression (TPM) of NASH-associated activated HMΦ genes. **(B)** Representative flow cytometry gating of HMΦ. Gated on CD45+ > CD11b+ Ly6G- > Ly6Clow MHCIIhigh. **(C)** F4/80+ mean fluorescence intensity (MFI) for HMΦ populations. Statistical Analysis: Bulk RNAseq analysis was performed using Qlucore Omics Explorer. PCA generated using significantly differentially regulated genes (p.adj < 0.01). Included genes were significant (p < 0.05) in multi-variant analysis (Two-Way ANOVA). Marked significance (*) determined by One-Way ANOVA. *p < 0.05, **p < 0.001, ***p < 0.0001 ****p < 0.0001. Flow cytometry data was analyzed with FlowJo, and statistical analysis was performed using Prism. One-Way ANOVA.

The results so far demonstrated that HFD promotes fatty liver in WT mice, however, caspase-11 deficiency decreases macrovesicular steatosis and lobular inflammation, which were well correlated with a report showing decreased macrophage recruitment into atherosclerotic lesion in Casp11^–/–^/ApoE^–/–^ atherogenic mice (109). Studies have shown that HMΦs drive inflammation and canonical pyroptosis in NAFLD, however, the role of noncanonical pyroptosis has not been well studied. Therefore, we next sought out to evaluate HMΦ pyroptosis. The transcripts of six NASH-associated activated macrophage markers including Cd5l, Clec4f, C1qb, Lpl, Folr2, and Il18bp were upregulated in both WT and Casp11^–/–^ mice (**Figure 3A**). Furthermore, we used flow cytometry analysis to examine liver macrophages (**Figure 3B**) and found that HFD promoted F4/80^+^ expression in HMΦs in both Casp11^–/–^ and WT mice (Figures 3C), indicating that HFD-induced NAFLD drives the increase of NASH-related F4/80^+^ HMΦs in WT mice, which are caspase-11 independent.

**Figure 4:**
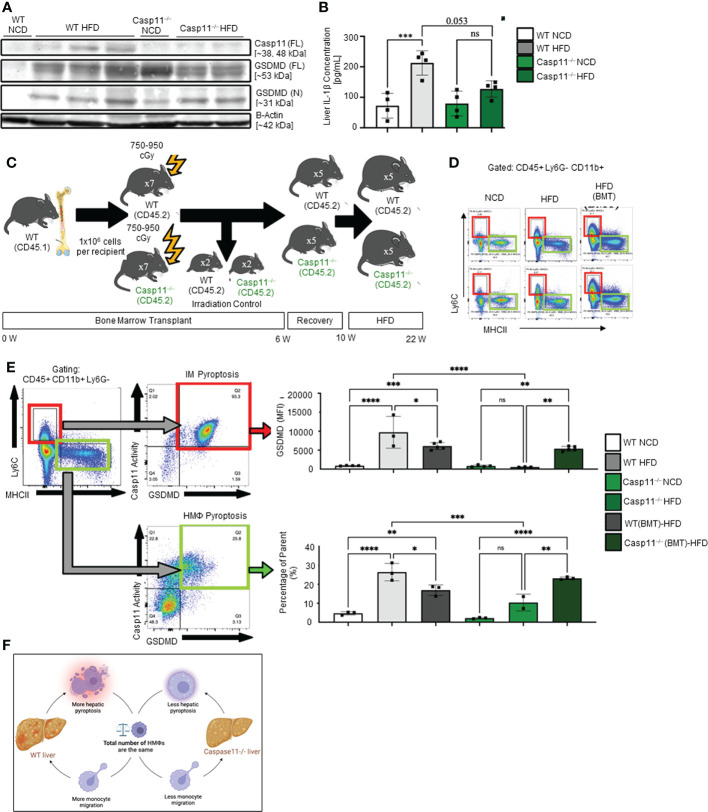
Hepatic inflammatory monocyte (IM) and monocyte-derived macrophage (MDM) caspase-11 deficiency protective against pyroptosis, WT bone marrow transplantation to Casp11^–/–^ mice restored IM and MDM pyroptosis. 8-10 weeks old male WT and Casp11^–/–^ mice were fed HFD for 12 weeks. **(A)** Western blot for noncanonical pyroptosis mediators. **(B)** Liver IL-1β concentrations. **(C)** Experimental design for bone marrow transplantation (BMT). **(D)** Representative flow cytometry gating of hepatic macrophages (HMΦ, Green, CD45+ > CD11b+ Ly6G- > Ly6Clow MHCIIhigh) inflammatory monocytes (IM, Red, CD45+ > CD11b+ Ly6G- > Ly6Chigh MHCIIlow). **(E)** Gating strategy for designing pyroptosis populations. HMΦs and IMs gated on GSDMD MFI vs. Casp11-Inhibitor MFI. RED: GSDMD MFI for IM (CD45+ > CD11b+ Ly6G- > Ly6Chigh MHCIIlow). GREEN: Percentage of the parent for HMΦ pyroptosis gating CD45+ > CD11b+ Ly6G- > Ly6Chigh MHCIIlow > Casp11 Activity vs GSDMD). **(F)** Schematic diagram showed that WT mice had more monocyte migration and more hepatic pyroptosis, however, Casp11–/– had less monocyte migration and hepatic pyroptosis resulting in an unchanged total number of hepatic macrophages. Statistical Analysis: Flow cytometry data was analyzed with FlowJo, and statistical analysis was performed using Prism. One-Way ANOVA. One-Way ANOVA. *p < 0.05, **p < 0.001, ***p < 0.0001 ****p < 0.0001. ns, Non-significant.

We examined caspase-11 and GSDMD expression in WT and Casp11^–/–^ HMΦs. We found that HFD increased caspase-11 and GSDMD expression in the inflammatory monocyte (IM) of WT mice while there were no significant changes in Casp11^–/–^ mice (**Figure 4A**). Liver expressions of IL-1β were significantly increased in WT mice upon HFD-feeding but the trending increase in IL-1β concentrations did not reach statistical significance in Casp11^–/–^ mice fed with HFD compared to Casp11^–/–^ mice fed with NCD (**Figure 4B**), suggesting that although caspase-1, other caspases (110), and neutrophil elastase (111) that are also capable of cleaving pro-IL1β are not deficient, HFD feeding induced IL-1β generation is mostly attributed by caspase-11 function. These results have demonstrated that HFD induced cytokine responses requires caspase-11.

Since 60% of mouse liver macrophages in disease conditions are derived from the bone marrow (25), to determine whether these changes were due to caspase-11 activity in bone marrow-derived macrophages, we performed bone marrow transplantation (BMT) (36). Bone marrow cells from WT donor mice (CD45.1) were transplanted into either WT recipient mice (CD45.2^+^) or Casp11^–/–^ mice after irradiation (**Figure 4C**). WT recipient mice (CD45.2^+^) that received BM from WT mice (CD45.1^+^) maintained significantly elevated GSDMD^+^ inflammatory monocytes (IMs) and noncanonical pyroptosis mature HMΦs (**Figures 4D, E**). Conversely, Casp11^–/–^ recipient mice (CD45.2^+^) that received bone marrow from WT mice (CD45.1^+^) had significantly regained GSDMD^+^ IM’s and noncanonical pyroptosis mature HMΦs (**Figures 4D, E**). These data indicate that WT mice had more monocyte migration and more hepatic pyroptosis, however, Casp11^–/–^ mice had less monocyte migration and hepatic pyroptosis presumably resulting in an unchanged total number of hepatic macrophages (**Figure 4F**). Taken together, our results have demonstrated that 1) caspase-11 deficiency significantly reprograms liver transcriptomes in NCD and HFD livers; 2) caspase-11 deficiency attenuates hepatic macrophage pyroptosis in HFD-induced NAFLD; 3) caspase-11 cleaves GSDMD-NT in NCD livers more than that in HFD-induced NAFLD; and 4) BM-derived macrophages play more significant roles than liver resident macrophages in developing and facilitating pyroptosis.

### 3.5 Caspase-11 deficiency significantly reduced extracellular acidification rates from glycolysis and mitochondrial electron transport chain functions suggesting that caspase-11 contributes to maintain dual fuel bioenergetics — glycolysis and oxidative phosphorylation in macrophages potentially for cholesterol biosynthesis

**Figure 5:**
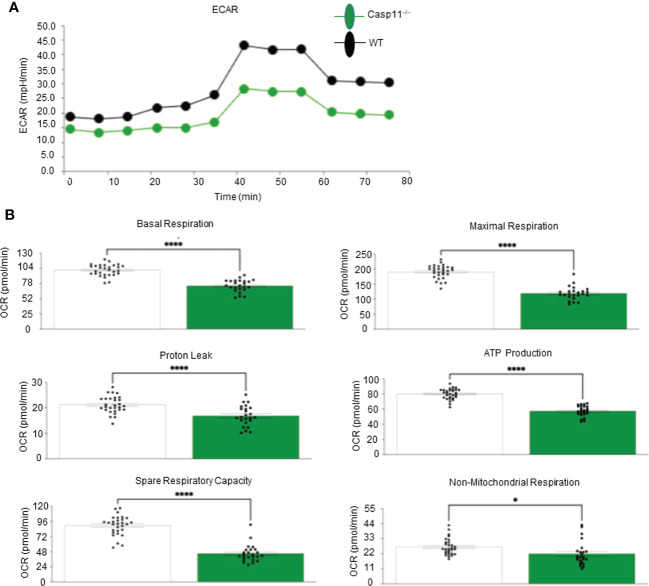
Caspase-11 significantly contributes to maintaining dual fuel bioenergetics- glycolysis and OXPHOS in macrophages potentially for cholesterol synthesis and trained immunity. Bone marrow macrophages were isolated from 3 WT and 3 Casp11^–/–^ mice and treated with palmitic acid (500 µM) for 8 hours then pulled for seahorse analysis. **(A)** Seahorse XF96 Extracellular Flux Analyzer to measure extracellular acidification rate (glycolysis) of Casp11^–/–^ vs WT BMDMs in palmitic acid supplemented medium. **(B)** Seahorse mitochondrial function assay of Casp11^–/–^ vs WT BMDMs in palmitic acid supplemented medium.*P < 0.0, ****p < 0.0001.

Our recent paper reported that two pathways such as fatty acid *β*-oxidation (112) and stearate biosynthesis are upregulated and shared by human NASH, NAFLD mouse models with glycine N-methyltransferase deficiency (GNMT-KO), and high-fat-cholesterol diet (HFCD) models (77). Our Casp11^–/–^ mice showed significant inhibition of NAFLD (**Figure 1**), implying that caspase-11 promotes cholesterol biosynthesis and fatty acid *β*-oxidation. We and others reported that increased acetyl-CoA promotes innate immune memory (trained immunity) (65, 88, 113–116); and fatty acid *β*-oxidation provides acetyl-CoA to fuel mitochondrial tricarboxylic acid (TCA) cycle and ATP production, which thus may not be limited to M2 macrophages (117). Previous reports showed that proinflammatory fatty acid palmitic acid induces hepatocellular lipotoxicity, endoplasmic reticulum (ER) stress, pyroptosis, and upregulate NLRP3 inflammasome, caspase-1 and IL-1β (118); and that caspase-11 deficiency leads to reduced activations of procaspase-1, IL-1β and caspase-7 and reduced production of glycolysis-promoted CXCL1 (119). In addition, caspase-11 may promote metabolic reprogramming and trained immunity (persistent hyperactivation of inflammation) (65, 113, 114, 120) as our transcriptomic data mining report suggested (77). One report supported this argument and showed that caspase-11 deficiency increases antimycin A-induced mitochondrial reactive oxygen species (mitoROS) (121–126) generation in macrophages (127), implying that caspase-11 inhibits mitochondrial electron transport chain (ETC) dysfunction and contributes the maintenance of mitochondrial ETC functions. We hypothesized that caspase-11 promote mitochondrial ETC functions in macrophages stimulated by palmitic acid. To examine the differences in the operation of mitochondrial energy pathways between WT and Casp11^–/–^ macrophages stimulated by NAFLD, gut derived endotoxins LPS, related (93, 128) proinflammatory saturated fatty acid palmitic acid using the method reported (93), we performed extracellular metabolic flux analysis. The extracellular acidification rates (ECAR) or proton efflux rate (PER), considered a proxy for glycolysis (129), were decreased in Casp11^–/–^ macrophages (**Figure 5A**). In addition, using the method we reported (87, 124–126), mitochondrial stress test results showed that six mitochondrial electron transport chain (ETC) functions including ATP production, maximal respiration, spare respiratory capacity (uncoupling of OXPHOS induced by carbonyl cyanide-p-trifluoromethoxyphenylhydrazone, FCCP), basal, proton leak and non-mitochondrial oxygen consumption were decreased in Casp11^–/–^ macrophages in comparison to that of WT macrophages in response to palmitic acid stimulation, especially ATP production, basal respiration, maximal respiration, and spare respiratory capacity (**Figure 5B**). Caspase-11 functions in maintaining both glycolysis and OXPHOS in macrophages stimulated by proinflammatory fatty acid palmitic acid are the same as that unique metabolic activation identified in adipose tissue macrophages (ATM) (130). The significance of the dual fuel bioenergetics in macrophages stimulated by hyperlipidemia and in adipose tissues may be related to an intermediate polarization status, their buffering capacity, or the result of a mixed population of distinctly polarized ATMs (131) and unique functions of caspase-11 in promoting HFD-induced NAFLD potentially by switching/transdifferentiating fatty acid *β*-oxidation -fueled OXPHOS in M2 macrophages into proinflammatory glycolysis-dominance in M1 macrophages (46). Taken together, these results have demonstrated that caspase-11 contributes significantly to the maintenance of glycolysis and mitochondrial electron transport chain functions in macrophages, in which acetyl-coenzyme A (acetyl-CoA) production is shared between glycolysis (acetyl-CoA transport into mitochondria) and TCA cycle (transport from mitochondria into cytosol for cholesterol synthesis); and both acetyl-CoA generation and cholesterol biosynthesis (132) are the key metabolic pathways for establishing trained immunity (69, 77, 114, 120), which are well correlated with our report on downregulation of 45.6% of 101 trained immunity pathway enzymes (71 glycolysis enzymes, 23 acetyl-CoA generation enzymes and 7 mevalonate synthesis enzymes) in Casp11^–/–^ transcriptome (GSE115094) (133) (77, 114).

## 4 Discussion

Nonalcoholic fatty liver disease (NAFLD) is the second leading cause of liver transplantation in the United States, and with obesity driven NAFLD on the rise worldwide, there is a great need for NAFLD research and therapeutic development (134, 135). Hepatic macrophage (HMΦ) activation and recruitment are important factors in driving the inflammatory phase of NAFLD and NASH (21, 47–50). While inhibition of macrophage activation and recruitment significantly decreases liver inflammation in NAFLD animal models, this may have unintended side effects due to the prevention of the physiological roles of HMΦs (18, 19, 31, 41–43). Therefore, a more targeted approach is required. Anti-inflammasome therapeutics have been shown to be a viable treatment option for inflammatory metabolic disease (72). While these therapies focus on the canonical pyroptotic pathway, the caspase11-dependent pyroptosis pathway provides a novel target and pathway for the treatment of NAFLD.

Based on our and other’s previous publications (77, 114, 136), HFD is one of the major drivers of innate immune memory (trained immunity). Glycolysis, Acetyl CoA generation (cytosolic and OXPHOS generated), increased mevalonate pathway, increased glutaminolysis, TCA cycle metabolite accumulation such as fumarate, and the epigenetic modification have all been identified as critical pathways for establishing trained immunity in trained immune cells. Furthermore, the proinflammatory cytokines including tumor necrosis factor-α (TNF- α), IL-1β, and IL-16 are the major read outs for trained immunity. In addition, inflammasomes control the maturation and secretion of proinflammatory IL-1β and IL-18 through GSDMD pores on the cell membrane and induce an inflammatory cell death (pyroptosis) (137). Therefore, the metabolic reprograming such as glycolysis in trained immune cells enhanced the release of IL1β through N-terminal GSDMD pores and promotes pyroptosis”.

The role of caspase-11 in driving pyroptosis in the pathogenesis of NAFLD in methionine- and choline-deficient diet (MCD)-induced NAFLD mouse model has been demonstrated (76). However, the role of caspase-11 in driving pyroptosis in HFD-induced NAFLD mice model have not been studied which we reported in this manuscript. Our team previously examined the expression changes of macrophage markers, macrophage proinflammatory cytokines, and macrophage metabolism genes in 10 macrophage subsets in liver inflammatory diseases, digestive inflammatory diseases, type-1 and type-2 diabetes, metabolic syndrome, and familial hypercholesterolemia and demonstrated that liver inflammatory diseases have predominant M1 macrophage status. In addition, M1 macrophage status have a significant upregulation of proinflammatory cytokine IL-1β, which secreted during pyroptosis mechanisms (138) and also reported in our recent papers (77, 139). We also found that M1 macrophages related to metabolic diseases have a significant increase in glycolysis while M2 macrophages related to TCA cycle metabolites (46). Therefore, ECAR and OXPHOS are related to cholesterol biosynthesis *via* increasing M1 macrophage glycolysis and increased generation of acetyl CoA which is the initial and key molecule for cholesterol biosynthesis as well as M2 macrophages which increases TCA cycle metabolites. IL-1 β is a major driver and regulator for innate immune memory (trained immunity) which characterized by increased glycolysis, increased acetyl CoA generation, and increased cholesterol biosynthesis (88, 140, 141).

Given the central role of HMΦs in the progression of NAFLD (21, 47, 49, 50) and the significance of pyroptosis in both patients and animal models of NAFLD outlined in our recent publication (77), we hypothesize that caspase-11-dependent pyroptosis promotes NAFLD *via* glycolysis and OXPHOS dual fuel bioenergetics and bone marrow-derived macrophage pyroptosis. We performed histopathological analysis, RNA-seq and scRNA-seq data analysis, FACS, Western blots, Seahorse mitochondrial stress analyses of macrophages and bone marrow transplantation on HFD-induced NAFLD in WT and Casp11^–/–^ mice, we made the following findings: 1) HFD feeding for 12 weeks drives increases NAFLD in WT mice, which are transcriptionally distinct from NCD control mouse livers; 2) Noncanonical pyroptosis mediators including caspase-11, GSDMD, IL-1β, and GBPs are increased in response to HFD; 3) HFD promotes type-I, secretome dominant, caspase-11-GSDMD pyroptosis other than type-II, inflammatory cell death-dominant, caspase-11-GSDMD pyroptosis; 4) Casp11^–/–^ mice have decreased NAFLD (reduced NAS score, steatosis score and lobular inflammation) with no significant liver weight changes; 5) Caspase-11 deficiency significantly decreases liver IL-1β concentrations and GSDMD expression; 6) Caspase-11 deficiency significantly reprogram liver transcriptomes in NCD and HFD livers, attenuates hepatic macrophage pyroptosis in HFD-induced NAFLD; 7) caspase-11 cleaves GSDMD-NT in NCD livers more than that in HFD-induced NAFLD; 8) bone marrow-derived macrophages play more significant roles than liver resident monocytes/macrophages in facilitating pyroptosis; and 9) Caspase-11 significantly contributes to maintain dual fuel bioenergetics — glycolysis and OXPHOS in palmitic acid-stimulated macrophages potentially *via* promoting transition of M2 macrophages into M1 macrophages.

**Figure 6:**
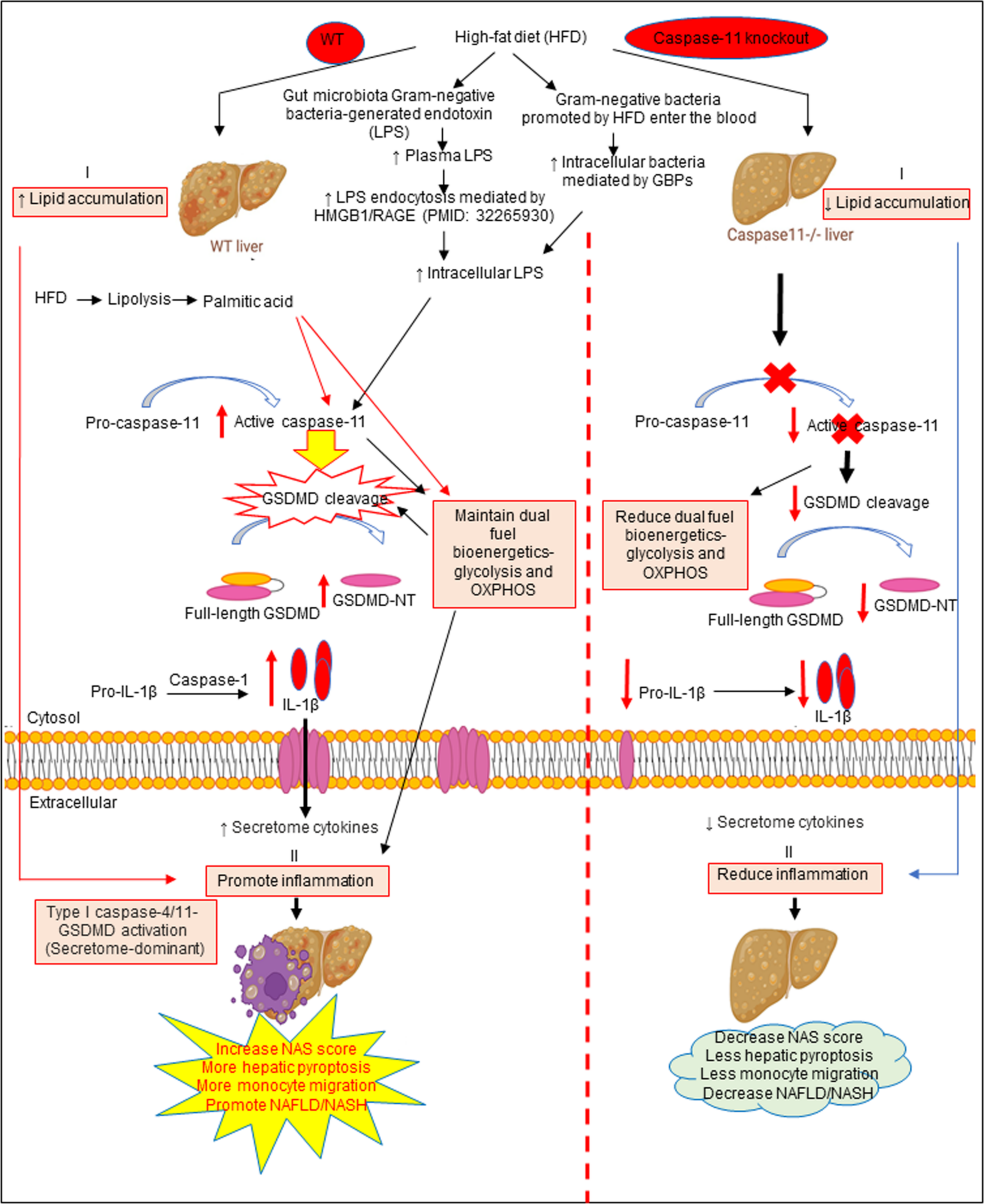
The working model showed that HFD increased hepatic lipid accumulation (steatosis). HFD promotes increased gut microbiota Gram-negative bacteria-generated endotoxin LPS leading to elevations in circulating LPS and metabolic endotoxemia and increased LPS endocytosis and intracellular LPS. Gram-negative bacteria promoted by HFD enter the bloodstream and enter cells which are mediated by GBPs to increase intracellular bacteria and LPS to activate caspase-11. Caspase-11 activation is triggered by its interaction with LPS from Gram-negative bacteria. Being an initiator caspase, activated caspase-11 functions primarily through its cleavage of key substrates. GSDMD is the primary substrate of caspase-11, and the GSDMD cleavage fragment generated (GSDMD-NT) leads to the formation of pores in the plasma membrane and secretion of caspase-1 produced IL-1B into the extracellular space to promote liver inflammation (NASH) and subsequently increased hepatic pyroptosis and promotes NAFLD. Thus, caspase-11 functions as an intracellular sensor for LPS and an immune effector. Palmitic acid produced by lipolysis in HFD-fed mice caspase-11 activation and GSDMD cleavage. Furthermore, LPS-induced caspase-11 activation and GSDMD cleavage also maintain dual fuel bioenergetics-glycolysis and OXPHOS and. Casp11^–/–^ decreased GSMDM cleavage and IL-1β secretion, reduced liver inflammation, and hepatic pyroptosis.

Based on our results, we propose a new working model; as shown in **Figure 6**, HFD increased hepatic lipid accumulation (steatosis). In addition, HFD promotes increase of gut microbiota Gram-negative bacteria-generated endotoxin LPS, leading to elevations in circulating LPS and metabolic endotoxemia (142), and increased LPS endocytosis (143) and cytosolic LPS. Furthermore, Gram-negative bacteria promoted by HFD enter the blood stream and enter cells, which are mediated by GBPs (144, 145) to increase intracellular bacteria and LPS to activate caspase-11. Caspase-11 activation is triggered by its interaction with LPS from Gram-negative bacteria. Being an initiator caspase, activated caspase-11 functions primarily through its cleavage of key substrates. GSDMD is the primary substrate of caspase-11, and the N-terminal GSDMD cleavage fragment generated (GSDMD-NT) leads to the formation of pores (protein channels) in the plasma membrane and secretion of caspase-1 produced IL-1β and other caspase-1 dependent secretomes and caspase-11-dependent secretomes (139, 146) into the extracellular space to promote liver inflammation (NASH), and subsequently increased hepatic pyroptosis and promotes NAFLD. Thus, caspase-11 functions as an intracellular sensor for LPS and an innate immune effector. Palmitic acid produced by lipolysis in HFD-fed mice activates caspase-11 and GSDMD cleavage. Furthermore, LPS-induced caspase-11 activation and GSDMD cleavage also maintain dual fuel bioenergetics — glycolysis and OXPHOS. Casp11^–/–^ decreases GSMDM cleavage and IL-1β secretion, reduces liver inflammation, and hepatic pyroptosis. These results provide novel insights on the roles of caspase-11-GSDMD pathway in promoting hepatic macrophage inflammation and pyroptosis and novel targets for future therapeutic interventions involving transition of NAFLD to NASH, hyperlipidemia, type-II diabetes, metabolic syndrome, atherosclerotic cardiovascular diseases, autoimmune diseases, liver transplantation, and hepatic cancers.

## Data availability statement

Our RNA sequencing data presented in the study are deposited in the NCBI’s Gene Expression Omnibus database repository, accession number GSE221005. Other RNA seq and single cell RNA seq data were obtained the NIH-NCBI-Gene database (https://www.ncbi.nlm.nih.gov/gene/837. Single Cell RNA-seq (scRNA-seq) Porter database from the MIT Broad Institute (https://singlecell.broadinstitute.org/single_cell/study/SCP1845/cross-tissue-immune-cell-analysis-reveals-tissue-specific-features-in-humans?genes=casp4&tab=distribution study-visualize were deposited in the ArrayExpress database at EMBL-EBI (www.ebi.ac.uk/arrayexpress) under accession number E-MTAB-11536).

## Ethics statement

The animal study was reviewed and approved by Institutional Animal Care and Use Committee (IACUC) and approved by the IACUC of Lewis Katz School of Medicine (LKSOM) at Temple University.

## Author contributions

CD and FS carried out the data gathering, data analysis and prepared the tables and figures. NJ, RC, YSu, KX, YSh, YL, HS, YZ, LY, JY, SW, NS, WH, JZ, YHZ, XJ, HW, aided with analysis of the data. XY supervised the experimental design, data analysis, and manuscript writing. All authors read and approved the final manuscript.
