# Supplementary material for: Caspase-11 promotes high-fat diet-induced NAFLD by increasing glycolysis, OXPHOS, and pyroptosis in macrophages
Source: Front Immunol. 2023 Jan 26;14:1113883. doi: 10.3389/fimmu.2023.1113883 (PMC9909353; doi:10.3389/fimmu.2023.1113883)
Supplement: Supplementary file 1 [file Presentation_1.pptx]

## Slide 1
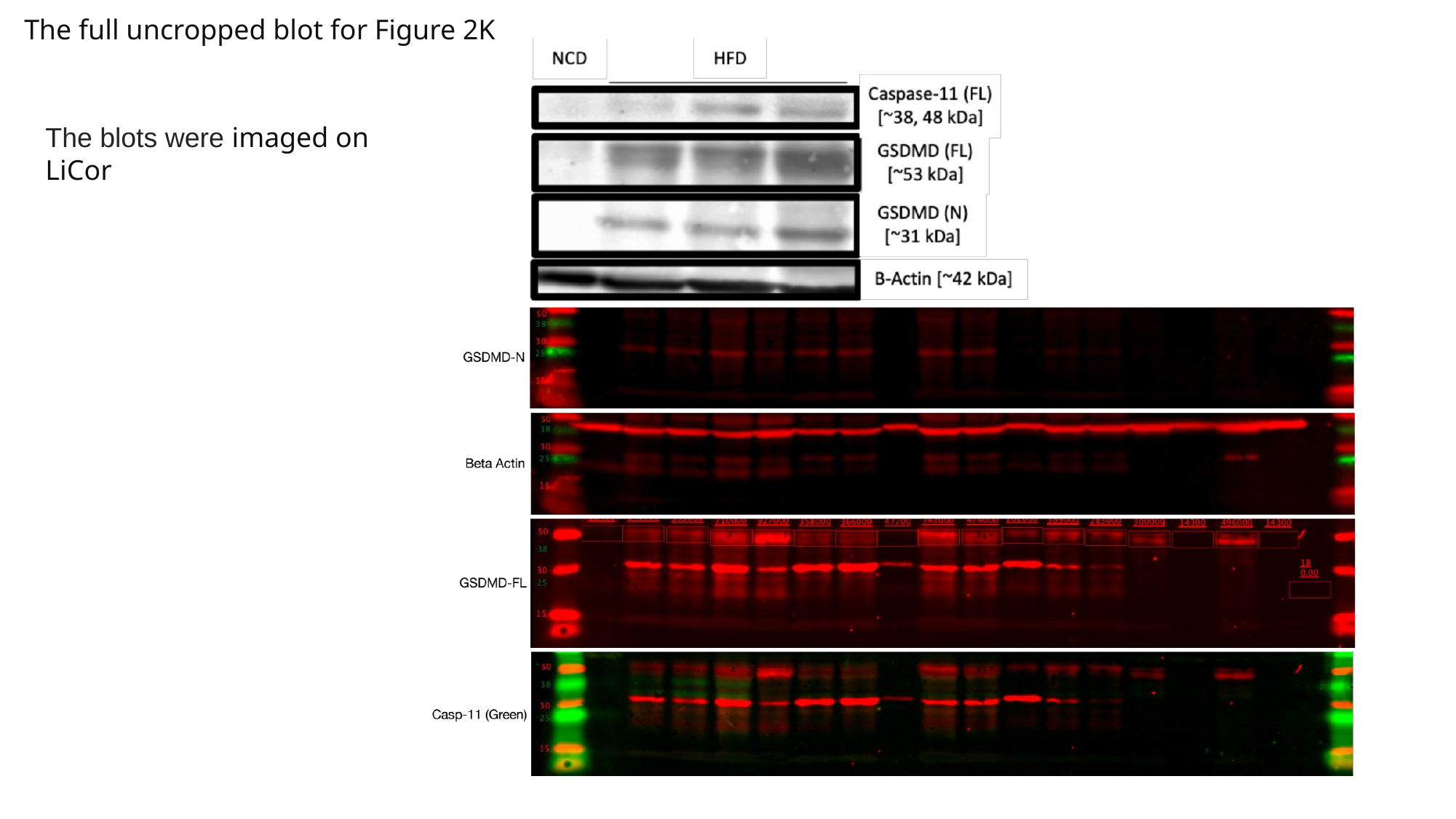

The full uncropped blot for Figure 2K
The blots were imaged on LiCor

## Slide 2
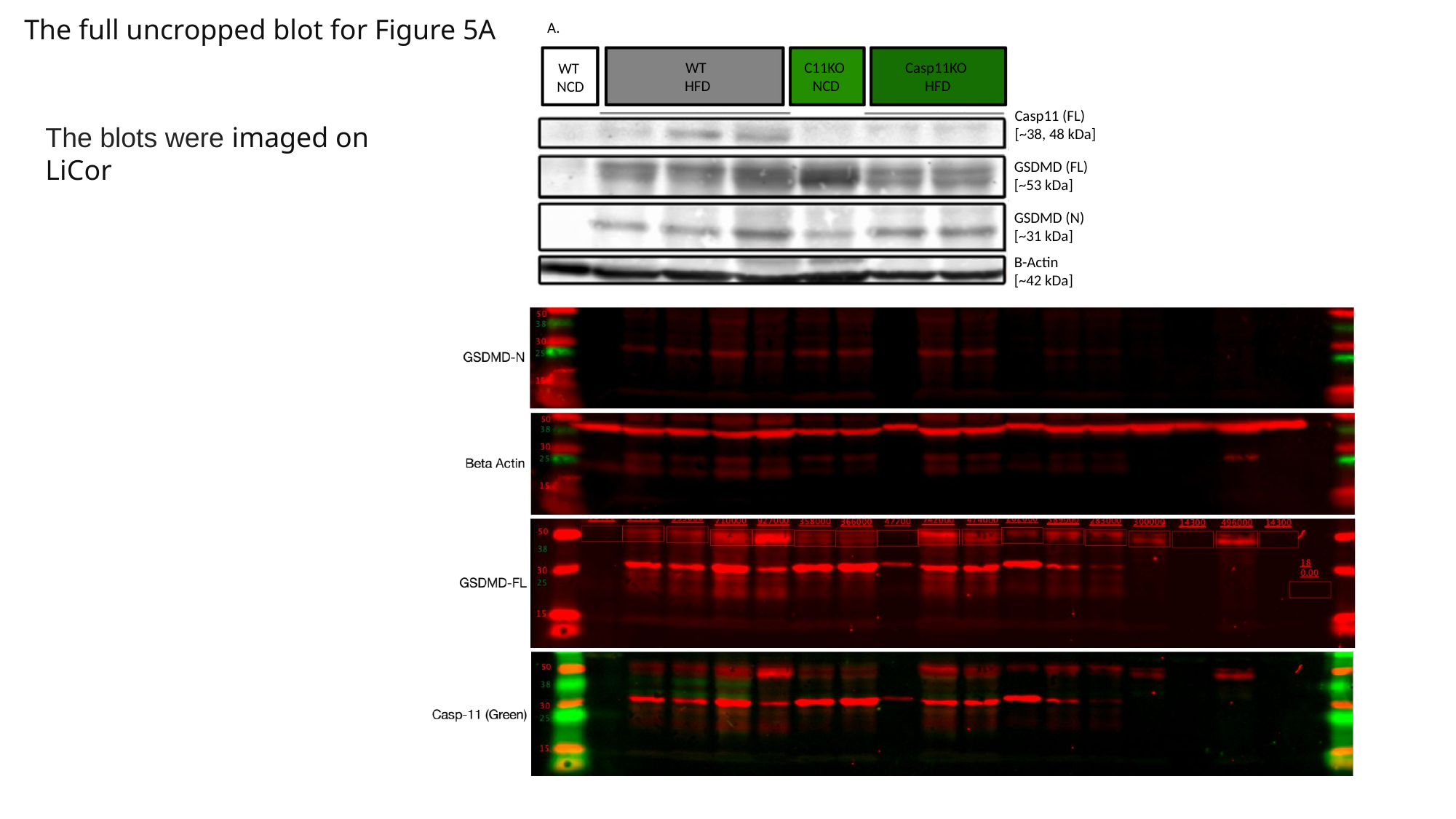

The full uncropped blot for Figure 5A
A.
WT
HFD
C11KO
NCD
Casp11KO
HFD
WT
NCD
Casp11 (FL)
[~38, 48 kDa]
GSDMD (FL)
[~53 kDa]
GSDMD (N)
[~31 kDa]
Β-Actin
[~42 kDa]
The blots were imaged on LiCor
